# Plant-based diets–impacts of consumption of little or no animal-source foods on human health

**DOI:** 10.3389/fnut.2024.1423925

**Published:** 2024-09-18

**Authors:** Alice V. Stanton

**Affiliations:** School of Pharmacy and Biomolecular Sciences, Royal College of Surgeons in Ireland, University of Medicine and Health Sciences, Dublin, Ireland

**Keywords:** animal-source foods, plant-source foods, plant-based diets, micronutrients, adequacy, non-communcable diseases

## Abstract

The world, in 2024, faces both climate and biodiversity crises, and the food system does contribute significantly to these crises. For some, the solution is simple - intakes of animal source foods (ASFs) should be considerably reduced, and consumption of plant-source foods (PSFs) should be greatly increased. Advocates for such a dietary transformation express confidence that plant-based diets will not only benefit planetary health, but will provide nutrient adequacy for all, and will also result in considerable protection from chronic non-communicable diseases (NCDs). However, as described in this perspective, the dramatic reductions in ASFs, entailed by many plant-based diets, will worsen already prevalent micronutrient and protein deficiencies. The protections provided by plant-based diets against NCDs appear to be more strongly associated with reduced intakes of calories and salt, and increased intakes of fruit, vegetables, nuts and whole grains, rather than with reduced intakes of ASFs. Any possible absolute adverse effects of red and processed meat consumption on NCDs are very small and uncertain. Other ASFs either appear to have no impact on NCDs (poultry meat and eggs), or are associated with protections against obesity, cardiovascular events, brain disorders and some cancers (seafood and dairy). Rigorous randomized controlled trials of all newly proposed environmentally-protective plant-based diets are required, so as to provide clear-cut evidence of micronutrient and protein adequacy, with or without, supplementation, fortification and/or biofortification. In the meantime, dietary guidelines should advise moderating excessive consumption, rather than substantially limiting or excluding ASFs from the human diet.

## Introduction

Humans have been omnivorous rather than herbivorous for a long time (1). About 3 million years ago, a period of climate change resulted in a decline of heavily forested lands, an expansion of drier grasslands and semi-forested regions, lessor availability of digestible plant source foods (PSFs), and greater availability of foods from grazing animals. Dietary divergence of hominins from other apes, toward animal source foods (ASFs), was followed by the physiological and metabolic adaptations that culminated in modern humans. With consumption of nutrient-rich, cooked, readily digested and absorbed ASFs, neither voluminous fermentation chambers, such as a rumen or cecum, nor an extensive colon, were required, gastrointestinal tract length and absorptive surface area could be greatly reduced, and brain size and complexity greatly increased (2).

However, the world in 2024, now faces both climate and biodiversity crises. Food production and consumption, and in particular livestock farming and consumption of its products, do contribute to these crises. The food system is currently estimated to be responsible for about one third of total greenhouse gas emissions (3), and the conversion of natural ecosystems to agricultural land has been reported to be the largest threat to species extinction (4). Hence there is indeed a need to transform our food system so that all have access to healthy diets, while at the same time safeguarding the planet’s health. The details of how that is best achieved is the subject of considerable debate – how much change should come from each domain of the food system – how much change should come from food production, processing, distribution, retailing, consumption and waste management?

For some, the solution to this challenge is simple, the human diet should revert back to being based on PSFs. It has been proposed that intakes of ASFs, particularly ruminant products, red meat and dairy foods, should either be considerably reduced, or totally excluded from the human diet (5–7). Advocates for such a dietary transformation express confidence that such plant based diets will not only benefit planetary health, but will provide nutrient adequacy for all, and will also result in considerable protection from chronic non-communicable diseases (cancers, diabetes mellitus, heart attacks and strokes).

In this article, the reliability of the claims of plant-based diets, with very reduced intakes of ASFs, for nutritional adequacy, and for protection against chronic disease events, is examined. Additionally, the impact and consequences of influential, but inaccurate, published metrics and recommendations, remaining uncorrected, are considered.

## Plant based diets – impacts of little or no ASFs on nutritional adequacy

In 2019 the EAT-Lancet Commission on Food, Planet and Health published their planetary health reference diet (5). This was probably the first attempt to balance human dietary and planetary environmental needs to generate widespread interest among nutritional and environmental scientists, health professionals, policy makers and the general public (8, 9). The paper made headlines across the world, and on social media, content connected to the report have had more than 1 million shares in over 200 countries (8). According to Altmetric, the report is among the top 20 most discussed science papers across all academia (9) – it has been cited by 5,593 scientific papers and 798 policy documents in the 5 years since publication.

The EAT-Lancet Commission’s planetary health diet is not a compulsory vegan diet – it does allow low quantities of red or processed meats and eggs to be consumed, and can include moderate amounts of seafood and poultry. However the diet largely consists of vegetables, fruits, whole grains, legumes, nuts and unsaturated plant oils – in total, only 13% of calories in the diet are from ASFs. Despite this low content of ASFs, the EAT-Lancet Commission were confident that the diet would meet all nutritional requirements of both adults and children older than 2 years. This confidence was surprising for a number of reasons.

Firstly, Beal and colleagues have clearly demonstrated that, as the percentage of energy coming from ASFs in national food supplies decreases, the prevalence of micronutrient inadequacy increases exponentially (10, 11). This particularly pertains to nutrients and micronutrients found in higher quantities, and in more bioavailable forms in ASFs, such as vitamins A, B_12_, and D, key minerals including calcium, iodine, iron, phosphorus and zinc, long-chain polyunsaturated omega-3 fatty acids (eicosapentaenoic acid and docosahexaenoic acid) and essential amino acids. Overall, Beal and colleagues concluded that an average of 35% of calories from ASFs is required to provide a nutritionally adequate diet for populations (10, 11).

A recently published systematic literature review of the subject has found clear-cut evidence that dietary changes aiming to reduce environmental impacts result in lower intakes and status of a wide range of micronutrients of public health concern (12). Most of the 56 studies included in this review suggested that folate intake would increase with plant-based diets, but intakes of zinc, calcium, iodine and vitamins A, B_12_ and D would all decrease. The review also reported that total intake of iron would increase, but that might not result in improved iron status due to the lower bioavailability of iron from PSFs.

The review relied primarily on observational and modeling studies – of the 56 included studies, 10 were dietary intake studies, 45 were dietary modeling studies, and only one was a randomized controlled trial with biomarker data. Pellinen et al. studied the effects of partly replacing animal proteins with plant proteins on vitamin B_12_, vitamin C, folate, iodine, iron and zinc, intakes and statuses in healthy adults (13). One hundred and 36 volunteers were randomly allocated to consume diets with 70% animal-source protein/30% plant-source protein, 50% animal-source protein/50% plant-source protein or 30% animal-source protein/70% plant-source protein, for 12 weeks. Key findings included that decreasing animal-source protein, even to the 50% level, led to important declines in the intakes and statuses of vitamin B_12_ and iodine. Zinc intake also decreased, but, due to the lack of an appropriate biomarker, zinc status was not evaluated. There were no differences in vitamin C intake nor status among the diet groups. While iron and folate intakes increased with greater consumption of PSFs, no significant differences in biomarker levels were observed. The authors concluded that longer duration trials, with biomarker data, in a range of healthy populations, were mandated to further study the effects of plant-based diets on the status of a wide range on nutrients, and particularly on iron status.

It is good that one of the EAT-Lancet Commissioners, Professor Jessica Fanzo, has recently confirmed that their first version of a planetary health diet would result in significant essential micronutrient shortfalls (14). In a paper published in Lancet Planetary Health in 2023, it was acknowledged that insufficient attention had been paid to the latest evidence on recommended nutrient intakes, to the greater bioavailability of iron and zinc from ASFs, and to the presence of anti-nutrients in many of the protein-rich PSFs. In the absence of micronutrient supplementation, in order to achieve micronutrient adequacy, it appears that intakes of ASFs, in such a flexitarian diet, would have to be doubled, accounting for at least 27% of calories, and intakes of PSFs, rich in phytates and polyphenols, such as whole grains, pulses and nuts, would need to be considerably reduced (14).

## Plant-based diets – impacts of little or no ASFs on chronic non-communicable diseases

In 2019, the EAT-Lancet Commission also expressed confidence that widespread uptake of their recommended diet would reduce the incidence of non-communicable diseases (NCDs) and overall mortality - they estimated that approximately 11 million premature deaths among adults could be avoided annually through global adoption of the diet (5). However, these estimates have not been universally confirmed in further modeling and observational studies.

Zagmutt and colleagues were the first to question these estimates of avoided mortalities – they identified flaws in the assumptions and methods used, and their corrected analysis suggested that any mortality reduction effect of the EAT-Lancet diet was no greater than the impact of energy consumption changes that would prevent under-weight, over-weight and obesity alone (15, 16).

Adherence to the EAT-Lancet reference diet was reported to be inversely associated with all-cause mortality in three reports, the United Kingdom Biobank Study (17), the Malmo Diet and Cancer Study (18), and in three prospective United States cohorts (Nurses’ Health Study I and II, and Health Professionals Follow-up Study) (19). It is noteworthy that the food groups contributing most strongly and consistently to the protection from mortality were increased intakes of PSFs rather than reduced intakes of ASFs – the top three food groups were fruits, vegetables and whole grains in the Swedish study (18), and added unsaturated fats, whole grains, and nuts in the United States study (19). A number of possible limitations were acknowledged by the authors of these three reports (17–19). Firstly, all cohorts were from high income countries. Secondly, those most adherent to the EAT-Lancet diet were also those most likely to follow a healthy lifestyle, and therefore residual confounding was highly likely to operate, and possibly explain some or all of the observed associations. Finally, adherence to the EAT-Lancet diet of even the most adherent subgroups was relatively low. The mean Planetary Health Diet Index score for the top decile in the United States-based cohorts was only 94 points out of a possible 140 points (19). Similarly, the dietary index of the quintile with highest adherence of the Swedish cohort ranged from 23 to 35 points out of a possible 42 points (18). In the United Kingdom Biobank study, the high adherence group did score 8 to 11 points out of a possible 11 points. However, due to lack of information in the United Kingdom Biobank questionnaire, adherence to three food groups (tubers, legumes and nuts) could not be assessed. Furthermore, this high adherence group accounted for less than 5% of the total cohort (17). Hence, the impact of strict adherence to the EAT-Lancet diet was, in reality, not tested in any of these three analyses.

By contrast, strict adherence to the EAT-Lancet reference diet was reported to provide no additional protection from mortality in the Oxford component of the European Prospective Investigation into Cancer and Nutrition study (20), the Prospective NutriNet-Santé Cohort study (21), and the Prospective Urban Rural Epidemiology (PURE) study (22). Interestingly, while adherence to the EAT-Lancet diet was not shown to be protective in the PURE study, adherence to the PURE healthy eating pattern was shown to be advantageous - each quintile higher PURE diet score was associated with a 9% (95% confidence intervals; 7–11) lower risk of death, and a 6% (3–8) lower risk of a major cardiovascular disease event (22). Rather than focusing on potentially disadvantageous foods, the PURE diet score is based on intakes of six protective foods, fruit, vegetables, nuts, legumes, fish and dairy (mainly whole-fat) (22). Hence, a key difference between the two diets is the guidance on ASFs. Intakes of meat (poultry, red and processed), dairy, fish and eggs should all be limited according to the EAT-Lancet diet (5). However, recent reviews have concluded that there is no additional risk of NCDs associated with consumption of poultry meat and eggs (23). Furthermore, based on evidence from cohort studies, metanalyses and biomarker studies of the protective effects of regular fish and dairy consumption against total mortality, cardiovascular disease, cognitive dysfunction, obesity and some cancers (7, 24–27), the PURE healthy eating pattern advises 2 to 3 servings of fish weekly, and 2 servings of dairy daily. An evaluation of the PURE diet score with and without each of the 6 food components confirmed that all 6 components, including the two ASFs, seafood and dairy, contributed to the observed protective associations. A further analysis of the PURE data found that inclusion of unprocessed red meat in the PURE score had no material effect on risk – hence, the PURE investigators did not advise any limitation to this food. This is in agreement with the conclusions of the comprehensive series of systematic reviews and guideline published in Annals of Internal Medicine in 2019 (28–34). The NutriRECs Consortium reported that the possible absolute effects of red and processed meat consumption on all-cause mortality are very small – reducing intakes of unprocessed red meat and processed meat by 3 servings weekly could prevent 8(0-15) and 9 (5–15) deaths per 1,000 persons, respectively, over 11 years (34). The consortium also, importantly, judged the certainty of evidence for this protection, as low or very low, and concluded that red and processed meat avoidance were not priority targets for improved human health (34).

The EAT-Lancet Commission relied on data and analyses from the Global Burden of Disease (GBD) 2017 Risk Factor Study (35) for their estimates of avoided mortalities achievable through global adoption of their diet. This GBD 2017 study reported that 11 million deaths (22% of all adult deaths), and 255 million disability adjusted life years (DALYs) (15% of all adult DALYs), were attributable to 15 dietary risk factors. High intake of sodium (3 million deaths and 70 million DALYs), and low intakes of whole grains (3 million deaths and 82 million DALYs), fruits (2 million deaths and 65 million DALYs), nuts and seeds (2 million deaths and 50 million DALYs), and vegetables (1.5 million deaths and 34 million DALYs) were the leading dietary risk factors. It is noteworthy that higher intakes of ASFs were estimated to be associated with protection against NCD events (seafood and dairy), or to have relatively small adverse impacts (unprocessed red meat: 25 thousand deaths and 1.3 million DALYs. processed meats: 0.1 million deaths and 3.6 million DALYs).

Using the above described GBD 2017 point estimates, the EAT-Lancet authors identified reduced intakes of salt, and increased intakes of whole grains, fruits, nuts and vegetables, as the main contributors to the putative planetary health diet’s protective effects. However, as previously highlighted by many leading nutritional epidemiologists, almost all nutritional variables are highly correlated with each other, and also with other lifestyle patterns (36, 37). The risk associations of excess salt consumption, and low intakes of whole grains, fruits, vegetables and nuts with disease burdens are neither independent, nor necessarily causal effects. Individuals with high intakes of calories, salt and ultraprocessed foods, are frequently the same individuals who rarely consume fruits, vegetables or oily fish, and who are also more likely to smoke and to take little exercise. Hence the GBD 2017 Diet Collaborators’ statement that dietary risks were responsible for 22% of all deaths and 15% of all DALYs among adults in 2017, very probably represents extensive residual confounding. Furthermore the use of causal language (“attributable to” and “responsible for”) by the GBD collaborators, when reporting on epidemiological associations, does not appear in accordance with good scientific principles (34, 36, 37).

The dangers of disregarding best practice in nutritional epidemiology (34, 36, 37), by using low-or very low-certainty evidence, in the development of guidelines, or in the calculation of global health metrics, is illustrated by the very different GBD risk estimates for unprocessed red meat, included in the GBD 2017, GBD 2019 and Burden of Proof (BoP) 2022 studies (35, 38, 39). In the 2017 estimates, based on associations with colorectal cancer and diabetes mellitus, the GBD Risk Factor Collaborators stated that diets high in unprocessed red meat were responsible for 25 thousand deaths and 1.3 million DALYs, globally (35). However, in 2019, the GBD Collaborators reported finding sufficient evidence supporting additional causal relationships of red meat intake with ischaemic heart disease, breast cancer, hemorrhagic stroke, ischaemic stroke and subarachnoid hemorrhage (38). Thus, they estimated that 896 thousand deaths and 23.9 million DALYs were attributable to unprocessed red meat consumption. This represented 36-fold and 18-fold increases over the GBD 2017 estimates for deaths and DALYs, respectively. The evidence for the 2019 estimates came from in-house, newly conducted, systematic reviews and meta-regressions - these had not been peer-reviewed nor published, and no assessments of certainty had been conducted. Many among the scientific community questioned the reliability of these dramatically changed estimates, and, rightly, requested publication of PRISMA (Preferred Reporting Items for Systematic Reviews and Meta-Analyses) compliant reports of the newly conducted systematic reviews (40–43).

These questions and requests eventually led to the publication of the BoP study of the health effects associated with unprocessed red meat consumption, in Nature Medicine in October 2022, by the GBD Collaborators (39). The relative risk curves and the conclusions of the BoP 2022 Study are very different from those of the GBD 2019 Risk Factors Study (38) - only the association between unprocessed red meat and colorectal cancer retained statistical significance. Even that relationship is doubtful, as statistical significance was only achieved after application of a monotonic constraint which resulted in an up to four-fold inflation of risk (44). In any case, the overall conclusion of the paper was similar to those of both the PURE study and the NutriRECS Consortium, namely that there is no or only very weak evidence that unprocessed red meat consumption is associated with any increased risk of NCDs.

## Consequences of delayed or non-correction of inaccurate metrics concerning ASFs and plant-based diets

The GBD collaborators have publically acknowledged that their 2019 risk estimates of unprocessed red meat for NCD events were erroneously greatly inflated (39, 45, 46). However, despite requests to the GBD authors, and to The Lancet’s editorial team and ombudsperson, no corrections have been applied to the published paper, and the 2019 risk estimates remain unchanged on the GBD website (47). Additionally, to date, the GBD collaborators have only published systematic reviews for the risk estimates associated with unprocessed red meat and with vegetable consumption (39, 48). No PRISMA compliant reports of the other 13 dietary risk factors have been published. Hence, considerable doubt remains over the accuracy of these GBD 2019 risk estimates.

Despite these important limitations, the GBD 2019 Risk Factors Study continues to be extensively cited. As can be seen in Table 1, the paper has been cited 3,651 times in the past 4 years. Among these publications, 233 have specifically commented on levels of red or processed meat consumption and/or its associated risks. At least 25 publications, in a wide range of national and international journals, have utilized the GBD 2019 Risk Factors Study’s theoretical minimum risk exposure level (TMREL) value of zero, and/or their relative risk curves, as the primary evidence for adverse outcomes being associated with, or caused by red or processed meat consumption (49–73). It is of concern that the monthly rate of such publications, using these erroneous estimates, continues to climb.

**Table 1:** Summary of the numbers of publications that have cited the GBD 2019 Risk Factors Study (November 2020 – Febuary 2024) over the past 4 years, and of the key findings of the 25 publications which have utilized the theoretical minimum risk exposure levels and/or the relative risk curves of GBD Risk Factors Study 2019 as primary evidence for adverse outcomes being associated with, or caused by, red or processed meat consumption.

Year	Total number of citations (number of citations/month)	Total number of citing publications mentioning levels of red or processed meat consumption and/or associated risks (number of citations/month)	Publications which utilized the theoretical minimum risk exposure levels and/or the relative risk curves of GBD Risk Factors Study 2019 as primary evidence for adverse outcomes being associated with, or caused by, red or processed meat consumption			
			First author	Title	Journal	Headline/Key Finding
2024	333 (167)	27 (14)	Hong et al. (49)	Global burden of diabetes mellitus from 1990 to 2019 attributable to dietary factors: An analysis of the Global Burden of Disease Study 2019	Diabetes, Obesity and Metabolism	The three largest dietary contributors to the burden of diabetes mellitus were high intake of red meat, high intake of processed meat, and low intake of fruit.
			Moreno et al. (50)	The burden of cardiovascular disease attributable to dietary risk factors in Australia between 1990 and 2019	PLoS ONE	Although the burden of diet-related CVD has decreased significantly in the Australian population over the past 30 years, diets low in wholegrains and high in red meat continue to contribute significantly to the overall CVD burden. Future nutrition programs and policies should target these dietary risk factors.
			Liu et al. (51)	Colorectal cancer’s burden attributable to a diet high in processed meat in the Belt and Road Initiative countries	World Journal Gastrointestinal Oncology	The burden of colorectal cancer in relation to the consumption of a diet high in processed meat threatens public health.
2023	1,576 (131)	97 (8)	Yan et al. (52)	Global burden of ischemic heart disease associated with high red and processed meat consumption: an analysis of 204 countries and territories between 1990 and 2019	BMC Public Health	Implementing targeted policies and interventions is required to reduce the burden of IHD caused by a high intake of red and processed meat.
			Liang et al. (53)	Distributions and Trends of the Global Burden of Colorectal Cancer Attributable to Dietary Risk Factors over the Past 30 Years	Nutrients	To alleviate colorectal cancer burdens, it is recommended to elevate the intake of whole grains, milk, calcium, and fiber while reducing consumption of red and processed meats.
			Sharma et al. (54)	Temporal patterns of breast cancer incidence, mortality, disability-adjusted life years and risk factors in 12 South American Countries, 1990–2019: an examination using estimates from the global burden of disease 2019 study	Breast Cancer Research and Treatment	Alcohol use, diet high in red meat and smoking contributed the maximum DALYs in most countries in 2019.			
			Li et al. (55)	Burden of early-onset colorectal cancer along with attributable risk factors from 1990 to 2019: a comparative study between China and other G20 countries	BMC Public Health	In China, the five leading risk factors, for both sexes, were diet low in milk [18.54% (95% UI: 12.71–24.07)], diet low in calcium [15.06% (95% UI: 10.70–20.03)], alcohol use [12.16% (95% UI: 8.87–15.64)], smoking [9.08% (95% UI: 3.39–14.11)], and diet high in red meat [9.08% (95% UI: 3.39–14.11)] in 2019.
			Forray et al. (56)	The Global Burden of Type 2 Diabetes Attributable to Dietary Risks: Insights from the Global Burden of Disease Study 2019	Nutrients	The results show that in 2019, 26.07% of T2DM mortality and 27.08% of T2DM DALYs were attributable to poor diets, particularly those low in fruits and high in red and processed meats.
			Wu et al. (57)	The Global Burden of Disease Attributable to Diet High in Red Meat in 204 Countries and Territories, 1999–2019: An updated Analysis of the Global Burden of Disease Study	Molecular Nutrition and Food Research	Globally, since 1999, deaths and DALYs caused by diets high in red meat have steadily increased.
			Romanello et al. (58)	The 2023 report of the Lancet Countdown on health and climate change: the imperative for a health-centred response in a world facing irreversible harms	The Lancet	Headline finding: In 2020, 7.8 million deaths were associated with insufficient consumption of nutritious plant-based foods and 1.9 million deaths were associated with excessive consumption of dairy, and red and processed meat.
			Mubarik et al. (59)	Breast cancer epidemiology and sociodemographic differences in BRICS-plus countries from 1990 to 2019: An age period cohort analysis	SSM - Population Health	High body mass index, high fasting plasma glucose, and a diet high in red meat contributed to the highest death and DALYs rates in most BRICS-plus nations in 2019.
			Zhang et al. (60)	Global Burden of Cardiovascular Disease from 1990 to 2019 Attributable to Dietary Factors	Journal of Nutrition	High socio-demographic index regions had the highest population attributable fractions for cardiovascular disease mortality and DALYs associated with high red and processed meat intake
			O’Hearn et al. (61)	Incident type 2 diabetes attributable to suboptimal diet in 184 countries	Nature Medicine	Largest type 2 diabetes burdens were attributable to insufficient whole-grain intake (26.1% (25.0–27.1%)), excess refined rice and wheat intake (24.6% (22.3–27.2%)) and excess processed meat intake (20.3% (18.3–23.5%))			
			Lv et al. (62)	Trend of disease burden and risk factors of breast cancer in developing countries and territories, from 1990 to 2019: Results from the Global Burden of Disease Study 2019	Frontiers in Public Health	Percentage changes in deaths from the seven risk factors in low-to middle-socio-demographic index regions increased significantly over time across all age groups. However, a diet with high red meat and high body mass index accounted for the most considerable increase in the magnitude.
			Wang et al. (63)	Trends of burden on ischemic heart disease and related risk factors among residents in Jiangsu Province, 1990–2019	Chinese Journal of Disease Control and Prevention	From 1990 to 2019, DALYs attributed to ambient particulate matter pollution (ARC = 1.29%), high body-mass index (ARC = 1. 76%), diet high in red meat (ARC = 0. 36%), diet high in processed meat (ARC = 0. 32%), and alcohol use (ARC = 4. 19%) exhibited the greatest increase.
2022	1,241 (103)	84 (7)	Liu et al. (64)	Worldwide burden attributable to diet high in red meat from 1990 to 2019	Archives of Medical Science	In 2019, a diet high in red meat was responsible for 0.9 million (95% UI 0.5 to 1.3 million) deaths and 23.9 million (95% UI 15.6 to 32.0 million) DALYs worldwide. From 1990 to 2019, the total deaths and DALYs attributable to a diet high in red meat increased by over 50%. Increasing consumption of red meat remains a global challenge, especially in the low-middle and middle SDI countries.
			Chen et al. (65)	Stroke mortality attributable to high red meat intake in China and South Korea: An age–period–cohort and joinpoint analysis	Frontiers in Nutrition	Controlling the intake of red meat may be a cost-effective strategy to reduce stroke mortality risk and the corresponding disease burden, especially for Chinese male individuals.
			Zhao et al. (66)	Epidemiological trends of female breast and gynecologic cancers in adolescents and young adults in China from 1990 to 2019: Results from the Global Burden of Disease Study 2019	Frontiers in Oncology	Of the deaths and DALYs, diet high in red meat was the greatest contributor to breast cancer, while a high body mass index was the greatest contributor to cervical, ovarian, and uterine cancers. A non-red meat diet, and the control of body weight could reduce female breast and gynecologic cancers burden in China.
			Li et al. (67)	Thirty-year changes in disability adjusted life years for colorectal cancer in China: a screening perspective analysis	Chinese Journal of Endemiology	Compared with 1990, the colorectal cancer -caused DALYs in China increased by 181.5% in 2019. Factors with the largest increase in the attributable percentage were high body mass index (151.1%), diet high in red meat (86.4%) and diet high in processed meat (78.8%).			
			Romanello et al. (68)	The 2022 report of the Lancet Countdown on health and climate change: health at the mercy of fossil fuels	The Lancet	Headline finding: in 2019, 1·9 million deaths were associated with excessive consumption of dairy, and red and processed meat.
			Chen et al. (69)	Long-Time Trend of Colorectal Cancer Mortality Attributable to High Processed Meat Intake in China and a Bayesian Projection from 2020 to 2030: A Model-Based Study	International Journal of Environmental Research and Public Health	Colorectal cancer death attributable to high processed meat intake is still high in China, and elderly males were at higher risk. Gradually decreasing the intake of processed meat could be an effective way to reduce colorectal cancer mortality.
			Wu et al. (70)	The burden of stroke attributable to risk factors and their trends from 1990 to 2019 in China	Chinese Journal of Disease Control and Prevention	From 1990 to 2019, the DALYs of ischemic stroke and intracerebral hemorrhage attributable to ambient particulate matter pollution, high BMI, alcohol use and diet high in red meat significantly increased by 410.46, 320.48, 277.03, 245.41 and 168.93%, 132.07, 60.01, 84.58%, respectively.
			Machado et al. (71)	Burden of non-communicable diseases attributable to dietary risks in Brazil, 1990–2019: an analysis of the Global Burden of Disease Study 2019	Revista da Sociedade Brasileira de Medicina Tropical	Diet high in red meat and sodium, and low in whole grains were the three main risk factors contributing to the burden of NCDs both in 1990 and 2019.
2021	501 (42)	25 (2)	Chung et al. (72)	Global red and processed meat trade and non-communicable diseases	BMJ Global Health	Results show that global increases in red and processed meat trade contributed to the abrupt increase of diet-related NCDs
			Romanello et al. (73)	The 2021 report of the Lancet Countdown on health and climate change: code red for a healthy future	The Lancet	Headline finding: between 2017 and 2018, estimated deaths due to excess red meat consumption rose by 1·8% to 842,000.

ARC, annual change rate. BMI, body mass index. BRICS, Brazil, Russia, India, China, and South Africa. CVD, cardiovascular disease. DALY, disability adjusted life-year. IHD, ischaemic heart disease. NCD, non-communicable disease. SDI, socio-demographic index. T2DM, type 2 diabetes mellitus. UI, uncertainty interval.

Two of these publications, the 2022 and 2023 Reports of the Lancet Countdown on Health and Climate Change (58, 68) used both the TMRELs of the GBD 2019 Risk Factors Study, and the optimal intakes of the EAT-Lancet Reference Diet, as evidence for their model assumptions concerning diet and health co-benefits. The headline findings of these two reports were similar - 11.5 million deaths were attributed to imbalanced diets, of which approximately 8 million deaths were associated with insufficient consumption of plant-based foods and 2 million deaths were associated with excessive consumption of dairy, red and processed meats. The reports’ estimates of 600,000 excessive deaths due to dairy consumption are particularly questionable – the authors assumed that the optimal intake for milk and dairy was zero to 250 mL per day, and stated that daily intakes above 250 mL contributed to overweight and obesity, and thereby caused approximately 600,000 cancer, cardiovascular or diabetic deaths annually. The authors appeared to ignore or disregard the already referenced evidence of two or more daily helpings of full-fat dairy (500–900 mL/day) being associated with protection against overweight, obesity and diabetes mellitus, colorectal and breast cancer, cardiovascular events and total mortality (7, 25–27).

**Figure 1:**
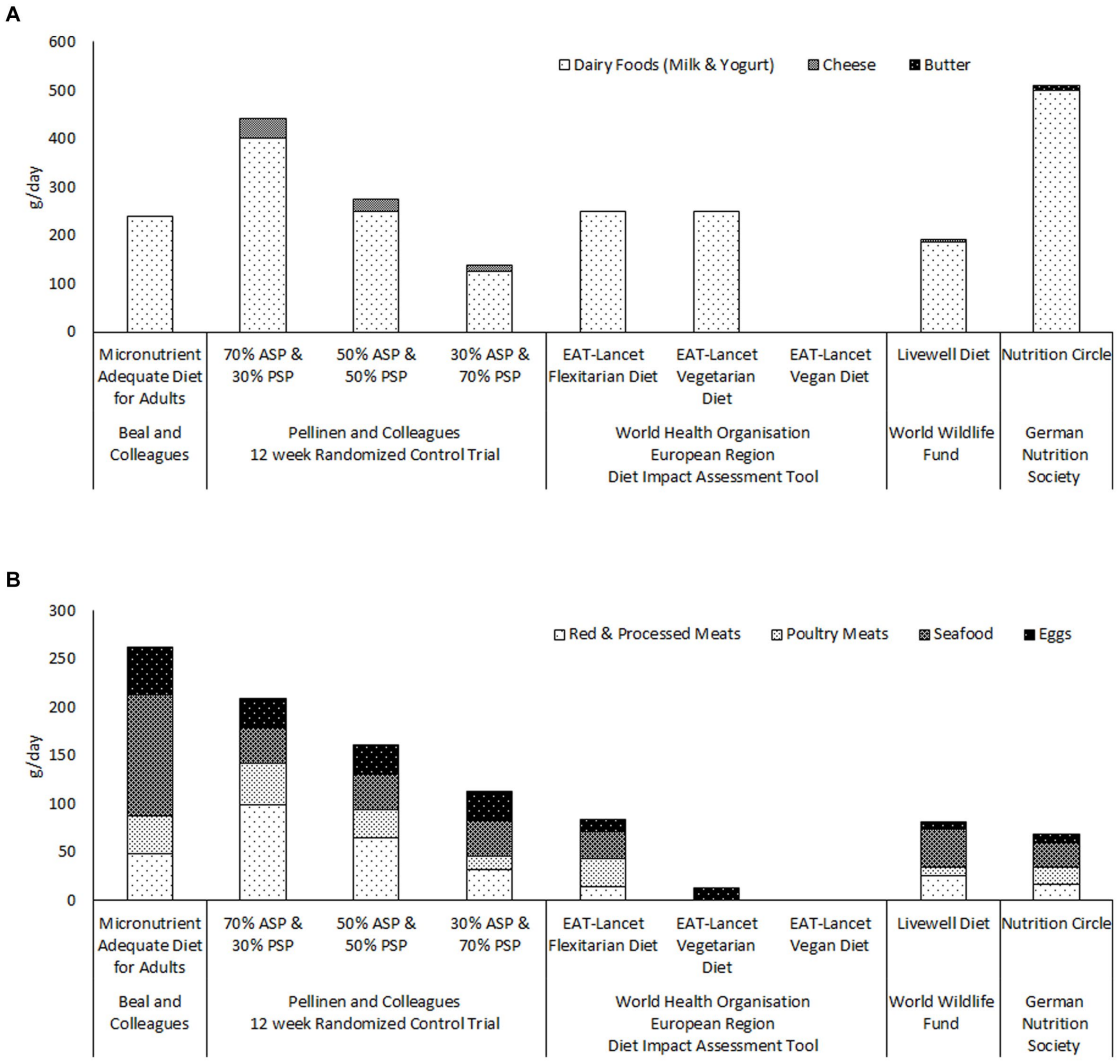
Comparison of the quantities of ASFs recommended by recently published guidelines for healthy and sustainable diets; the World Health Organisation European Region’s Flexitarian, Vegetarian and Vegan diets (7); the World Wildlife Fund’s Livewell diet (75); and the German Nutrition Society’s Nutritional Circle (74), with the quantities included in Beal and colleagues’ Micronutrient Adequate Diet for Adults (14), and in the three food groups of Pellinen and colleagues’ randomised controlled trial (13). Panel **(A)** illustrates the quantities of dairy foods recommended by each of the diets. Panel **(B)** illustrates the quantities of meats, seafood and eggs recommended by each of the diets. ASP; animal-source protein. PSP; plant-source protein.

The reports from the EAT-Lancet Commission and the GBD Risk Factors Collaborators also appear to continue to influence food policy decisions and international dietary guidelines. Figure 1 illustrates the quantities of ASFs recommended by a number of recently published international and national guidelines for healthy and sustainable diets (7, 74, 75). Only the German Nutrition Society (74) recommends two servings of dairy per day (Figure 1A, panel). The maximum dairy intakes recommended by either the World Health Organization (WHO/Europe) (7) or the World Wildlife Fund (75) is one serving per day ( ≤250 ml/day). It is noteworthy that the WHO/Europe diet impact assessment tool uses the same models to evaluate human health impacts as the above described reports of the Lancet Countdown on Health and Climate Change Commission (58, 68) Figure 1B panel illustrates that the total amounts of meat, seafood and eggs, recommended by World Health Organization, the World Wildlife Fund and the German Nutrition Society, are less than a third of the total required for micronutrient adequacy according to Beal and colleagues (14). Indeed the quantities of meat, seafood and eggs recommended by the five diets, from these three bodies, are all less than those consumed by the 30% animal-source protein group of Pellinen and et al. randomized controlled trial (13). It is difficult to see how any of these diets could provide either protein or micronutrient adequacy at the population level.

## Concluding comments

It is clear that any evidence that moderate consumption of ASFs is detrimental to human health, is weak and uncertain. The relationship between red meat and disease burden, like those of calories and salt with disease burden, is most likely U-shaped. Excess red and processed meat consumption (>4 portions or 500 g/ week) may be associated with very small increases in morbidity and mortality (low certainty evidence). Insufficient meat consumption (<2 portions/week) is associated with very large increases in anemia, stunted childhood growth and cognition, osteoporosis and sarcopenia (high certainty evidence). Poultry meat and eggs appear to have no impact on NCDs, while consumption of dairy and seafood not only protects against key deficiencies, these foods also likely protect against obesity, cardiovascular events, brain disorders and some cancers.

It is also clear that the dramatic reductions in ASFs, advised by many plant-based diets, will worsen already prevalent micronutrient and protein deficiencies worldwide. This will have particular impact in low and middle income countries, and on vulnerable groups, including women, children and the elderly. These were the conclusions of Ty Beal’s recent editorial in the American Journal of Clinical Nutrition (76). I agree with his calls for; moderating excessive consumption, rather than substantially limiting or excluding ASFs from the human diet; and further research into the roles that supplementation, fortification and biofortification can play in achieving healthy sustainable diets for all. Furthermore, it is of considerable importance that rigorous randomized controlled trials of all newly proposed environmentally protective diets are conducted. These trials should include validated biomarkers of nutrient status, and should assess levels of supplementation and/or fortification, that would be required so as to ensure micronutrient and protein adequacy.

Finally, scientists, policy-makers and all involved in the food system should be extremely wary of reports, guidelines or global health estimates that are not rigorously and transparently evidence-based. A wide range of sustainably produced, nutrient-rich, animal-and plant-sourced foods, in appropriate evidence-based quantities, should continue to be included in national and international guidelines for healthy diets. Further research, finances and effort should be directed toward objective and reliable measurements and improvements in sustainability of each component of the food system; production; processing; distribution; retailing; consumption; and waste management.

## Data Availability

The original contributions presented in the study are included in the article, further inquiries can be directed to the corresponding author/s.
